# Effect of Myostatin Gene Mutation on Slaughtering Performance and Meat Quality in Marchigiana Bulls

**DOI:** 10.3390/ani12040518

**Published:** 2022-02-19

**Authors:** Simone Ceccobelli, Francesco Perini, Maria Federica Trombetta, Stefano Tavoletti, Emiliano Lasagna, Marina Pasquini

**Affiliations:** 1 Dipartimento di Scienze Agrarie, Alimentari ed Ambientali, Università Politecnica delle Marche, 60131 Ancona, Italy; m.f.trombetta@staff.univpm.it (M.F.T.); s.tavoletti@staff.univpm.it (S.T.); m.pasquini@staff.univpm.it (M.P.); 2 Dipartimento di Scienze Agrarie, Alimentari e Ambientali, University of Perugia, 06121 Perugia, Italy; francesco.perini@studenti.unipg.it (F.P.); emiliano.lasagna@unipg.it (E.L.)

**Keywords:** *GDF8*, beef cattle, productive performance, gene polymorphism

## Abstract

**Simple Summary:**

The aim of this work was the evaluation of slaughtering performance in a sample of 78 Marchigiana bulls with different allelic situation at the myostatin locus; in addition, the qualitative composition of meat samples collected from *Longissimus thoracis* muscle was evaluated. At the myostatin gene, 67 homozygotes normal, 11 heterozygotes, and no double-muscled homozygote bulls were detected. Heterozygote bulls showed high values in final live weight and dressing yield; moreover, they were characterized by a low incidence of fat at steak dissection, as well as in meat chemical composition. A better muscular conformation in heterozygote bulls’ carcasses was highlighted, with a higher incidence of their carcasses in class E and evident convexity of round, back, and shoulder muscular masses compared to the carcasses of Marchigiana bulls which were normal at the myostatin gene.

**Abstract:**

The myostatin gene also called Growth Differentiation Factor 8 gene (*GDF8*) is one of the most investigated *loci* that can be responsible for several quantitative and qualitative carcass and meat traits in double-muscled beef cattle. The objective of the study was to bring to light the effect of the myostatin polymorphism on slaughtering performance and meat quality in Marchigiana beef cattle. The experiment was carried out on 78 bulls reared according to the “cow-calf” extensive managing system. At the end of the fattening period, in vivo and carcass data were recorded. From each carcass, a steak of *Longissimus thoracis* was taken and used to determine the meat’s analytical composition and colorimetric properties. Finally, from each steak a sample of *Longissimus thoracis* was collected, then used for DNA extraction and genotyping at the myostatin locus. The heterozygous bulls showed slight superiority in the carcass data (e.g., hot carcass weight: 426.09 kg—heterozygotes vs. 405.32 kg—normal) and meat quality parameters, although not always with statistical significance. Only fat and ashes content were significantly affected by the myostatin genotype (heterozygotes: 2.01%, 1.26%; normal: 3.04%, 1.15%). The greater muscularity of heterozygous animals compared to normal ones could be a starting point to improving productive efficiency in Marchigiana beef cattle.

## 1. Introduction

Increasing food demand will lead to the application of more efficient agricultural technologies. In this perspective, the meat industry would gain an advantage from strategies aimed at improving the efficiency of beef cattle, such as genetic selection, management practices, rumen functional efficiency, and structure and composition of feed [1]. In addition, the growing consumer demand for lean meat with low levels of fat has become one of the main targets of the beef cattle production chain [2], although there is also an opposite trend, with consumers preferring beef characterized by variable fat content leading to more intense flavor.

In Italy the meat market is changing, following modern consumers’ needs and new economic dynamics [3]. Knowledge of carcass and meat quality is pivotal for the beef market [4], and crucial for the future of many beef production systems. Consequently, it is of great importance to deepen the knowledge on the effects of contributing genes to explain variability related to carcass and meat quality traits in beef cattle breeds [5].

The myostatin (*MSTN*) gene, also called Growth Differentiation Factor 8 gene (*GDF8*), has been recognized as important in the development of the skeletal muscles and, hence, in animal growth and carcass traits [6,7,8]. Genetic variation in *MSTN* has been identified in different vertebrate species [9,10,11]. More than 20 different mutations (deletions, insertions, and nucleotide substitutions) have been reported in bovine *MSTN* [12]. Nucleotide changes result in whole or partial loss of function of myostatin activity and consequently in the known double-muscled phenotype [13]. Bovine muscular hypertrophy has extended widely among several European cattle breeds, leading to greater growth rates and carcass value [14]. In many breeds where the phenotype appears, the hypertrophy displays differences on frequency distribution, probably due to changes in selection pressure, which differ depending on market and management requirements [15]. Moreover, extreme muscle hypertrophy is sometimes undesirable by breeders, depending on some problems that can affect the hypertrophied animals (which include macroglossia, hypoplasia of vital organs, high instances of dystocia), and therefore, the genetic management of muscular hypertrophy can differ among different breeds and countries [16].

The Marchigiana is one of the most important Italian beef cattle breeds, and its fresh meat, since 1998, can be certified by the Protected Geographical Identification (PGI) “Vitellone Bianco dell’Appennino Centrale” [17]. The selection program is carried out by the National Association of Italian Beef Cattle Breeders (ANABIC), and the current breeding goal is based on appreciable improvement of its performance for meat-related traits [5]. Marchigiana cattle may also show muscular hypertrophy, due to a mutation at nucleotide 874 in exon III (g.874G > T) in the *MSTN* gene. This mutation (E291X variant) affects the myostatin protein due to the introduction of a premature stop codon, thus blocking the translation of 254 bases of the third exon. Myostatin is a negative regulator of muscle mass development which suppresses both the proliferation and differentiation of myogenic cells. The dysfunctional myostatin leads to an increase in muscle mass in animals carrying the causative mutation. Therefore, animals can have normal (G/G) or hypertrophied (heterozygous G/T or homozygous T/T) genotypes [12]. Double-muscled carcasses generally have high dressing percentage, ranging from 64 to 67% [18], large dimension of muscles, and low proportion of fat and bone [19,20], making these traits economically interesting in the Marchigiana breed.

To the best of our knowledge, this is the first report focused on performance and key meat quality parameters in heterozygous Marchigiana bulls at the *MSTN* gene. Thus, the objective of the present study was to verify and quantify the effect of the *MSTN* polymorphism on both slaughtering performance and meat quality in Marchigiana beef cattle.

## 2. Materials and Methods

### 2.1. Animals

The study was carried out on 78 Marchigiana bulls reared according to the “cow-calf” extensive managing system [21], which is traditional of the Marche region (Italy). The animals were all registered in the Herd Book and were unrelated based on their pedigree information. The animals were harvested using standard commercial procedures at the commercial harvesting facility of BOVINMARCHE [22]. During the indoor fattening period, carried out in the same local livestock farm, bulls were fed ad libitum ration, based on first cut hay from local fodder crops, and a farm-made mixture concentrate (corn, barley, and faba bean), usually administered top-dressed to the forage portion once or twice a day (30:70 as Forage:Concentrate ratio). Ration was sampled monthly and analyzed for the analytical composition of dry matter (DM) (Table S1) according to AOAC official methods [23].

### 2.2. In Vivo and Carcass Data

At the end of the fattening period (120 ± 30 days), the final live weight (FLW) of each animal was recorded, then animals were slaughtered in a commercial abattoir according to European guidelines [24]. The average daily gain (ADG) was computed as final live weight/number of lifetime days. After slaughtering, hot carcass weight was recorded (HCW) with an electronic balance to evaluate carcass daily gain (CDG = HCW/number of lifetime days) and dressing yield (DY = (HCW/FLW) × 100). An assessor performed the classification for carcass profiles’ conformation (SEUROP system with six classes: S—superior, E—excellent, U—very good, R—good, O—fair, P—poor) and fat cover (with 5 classes: 1—low, 2—slight, 3—average, 4—high, 5—very high) on each carcass, according to European legislation [25]. The pH measurement at 45 min post mortem (pH-45′) was performed on *Longissimus thoracis* (LT) muscle between the 8th and 9th thoracic vertebrae using a portable pH-meter (pH110, Eutech Instrument, Thermo Fisher Scientific, Massachusetts, USA) equipped with the automatic temperature compensation function and a food probe (Food-Trode 120, XS sensor, Carpi, Italy) calibrated with pH = 7 and pH = 4 buffers prior to measurements.

### 2.3. Meat Analytical Composition and Colorimetric Properties

About 24 h after slaughtering, one steak was sampled from each carcass (78 total steaks) between the 8th and 9th thoracic rib, transferred to the laboratory under refrigerated condition (4 °C) for further meat evaluations. Firstly, steaks’ colorimetric profiles were evaluated. Chroma Meter CR-200 (Minolta, Tokyo, Japan) was used to detect the colorimetric profile of Marchigiana meat samples, determining the coordinates of lightness (L*: 0 = black; 100 = white), redness-greenness (a*: + red; − green), and yellowness-blueness (b*: + yellow; − blue) according to the CIE Lab color space system with illuminant D65, 2° observer, Diffuse/O mode, 8 mm aperture of the instrument for illumination, and 8 mm for measurement [26]. The colorimeter was standardized with a white tile (L* = 97.14, a* = − 0.61 and b* = 2.75). Moreover, the chromaticity of meat was evaluated as chroma (C), which is considered the quantitative attribute of colorfulness, using the equation √ (a^2^ + b^2^). The colorimetric readings were performed in triplicate for each sample.

Afterwards, each steak was weighed with electronic balance (accuracy ± 1.0 g, Entris^®^ II Advanced Line, Sartorius, Goettingen, Germany) and dissected to determine the percentage of bone, LT muscle, other muscles, and fat.

Then, drip loss was measured to evaluate meat water-holding capacity in LT muscle [27]. Initial samples’ weights were recorded; after a period of 24 h at 4 °C, samples were removed from plastic bags, gently blotted dry, and weighed again. Drip loss was expressed as a percentage ((weight after drip/initial sample weight) × 100). The remaining LT muscle was then frozen and lyophilized using a VirTis Advantage Lyophilizer (VirTis SP Scientific, Gardiner, NY, USA) to calculate moisture content and used to determine, in duplicate, meat compositional parameters. Protein (Kjeldhal method), fat (extraction with petroleum ether), and ash percentages (incineration in muffle furnace at 550 °C) were performed according to AOAC official methods [23].

### 2.4. Myostatin Genotyping

A sample of 50 g of LT from each steak was taken, then lyophilized using the method previously described, and stored at −20 °C until DNA extraction was performed. Genomic DNA was extracted using the GenElute Tissue Genomic DNA kit (Sigma–Aldrich, St. Louis, MO, USA) following the specific manufacturer protocol. The genotype of all animals was determined by PCR-RFLP (Polymerase Chain Reaction-Restriction Fragment Length Polymorphism) following a modified method developed by Marchitelli et al. [28]. The determination of the genotype imposes a first amplification of a portion of the exon III containing the point mutation at nucleotide 874 bp. The sequences of primers [29] used for amplification were: 5′-TGAGTCCTTGAGGTAGGAGAGTG-3′ (forward) and 5′-GGGGAAGACCTTCCATGTTT-3′ (reverse). The expected amplification product is a fragment of 448 bp.

PCR amplification was performed in 25 μL reactions containing 50 ng of genomic DNA as template, 3 mM MgCl_2_, 50 mM of each dNTP, 1 mM of each primer, and 1 unit of Taq^®^ DNA Polymerase (Sigma–Aldrich, St. Louis, MO, USA), using a TGradient 96 Thermocycler (Biometra GmbH, Göttingen, Germany). The PCR conditions were as follows: initial denaturation step of 5 min at 94 °C, 30 cycles of 30 s at 94 °C, 30 s at the 57 °C, 1 m at 72 °C, and a final extension of 5 min at 72 °C.

The PCR product underwent the digestion with restriction enzyme Tru9I (Thermo Fisher Scientific Inc., Waltham, MA, USA). A total volume of 20 µL contained 300 ng of amplicon, 1X Buffer R (10 mM Tris-HCl (pH 8.5), 10 mM MgCl_2_, 100 mM KCl, 0.1 mg/mL Bovine Serum Albumin—BSA), and 1 U of Tru9I. The digestion conditions were set at 65 °C for 1 h. Finally, the genotypes for all samples were assessed through a 2.5% agarose gel electrophoresis and then recorded.

### 2.5. Statistical Analysis

Data were analyzed by analysis of variance and covariance (ANOVA and ANCOVA), and multiple comparisons among means (JMP 11.0 software, SAS Institute, Cary, NC, USA). Before performing the statistical models, normal distribution of residuals and homogeneity of variances were tested according to the Shapiro–Wilk and Barlett tests, respectively. Seven animals were excluded from the carcass data analysis because they were outliers for the length of the fattening period.

For the analysis of final live weight (FLW), average daily gain (ADG), hot carcass weight (HCW), carcass daily gain (CDG), dressing yield (DY), carcass pH 45 min post mortem (pH-45′), and dissection variables, the following fixed model ANCOVA was applied:

(1) $$y_{\mathrm{ij}}=\mu +{\;G}_{i}+b\left(X_{\mathrm{ij}}-\;\bar{X}\right)+{\;\varepsilon }_{\mathrm{ij}}$$

where y_ij_ was the dependent variable for the j-th animal, µ was the overall mean, G_i_ was the fixed effect of genotype (i = 1, 2; normal or heterozygous at the *MSTN* gene), and b was the regression coefficient of the covariate: age at slaughtering (X) for FLW, ADG, HCW, CDG, and DY; hot carcass weight (X) for pH-45′ and dissection variables (bone%, LT muscle%, other muscle%, and fat%). The term ε_ij_ was the residual error.

For the analysis of drip loss percentage, the following fixed model ANOVA was applied:

(2) $$y_{\mathrm{ij}}=\mu +{\;G}_{i}+{\;\varepsilon }_{\mathrm{ij}}$$

where y_ij_ was the dependent variable for the j-th animal, µ was the overall mean, G_i_ was the fixed effect of genotype (i = 1, 2; normal or heterozygous at the *MSTN* gene), and ε_ij_ was the residual error.

For the parameters of meat composition and meat colorimetric indexes, double and triplicate measurements were respectively performed for each steak. Therefore, the following fixed ANOVA model, including the sampling error, was applied to the complete data set:

(3) $$y_{\mathrm{ij}}=\mu +{\;G}_{i}+{\;\varepsilon }_{\mathrm{ij}}+{\;\delta }_{\mathrm{ijk}}$$

where y_ij_ was the dependent variable for the j-th animal, µ was the overall mean, G_i_ was the fixed effect of genotype (i = 1, 2; normal or heterozygous at the *MSTN* gene), ε_ij_ was the experimental error, and δ_ijk_ was the sampling error.

Student’s *t*-Test was performed to compare the least square means of the two genotypes at the *MSTN* locus. The χ^2^ test was applied to test the Hardy–Weinberg equilibrium for the *MSTN* locus and to compare the frequencies at the SEUROP grid and fat cover categorical classes of the two genotypes at the *MSTN* gene.

## 3. Results and Discussion

**Figure 1:**
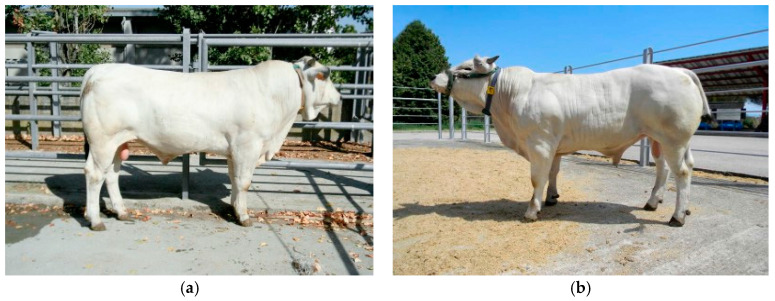
Representative pictures of the phenotype in Marchigiana breed: (**a**) normal bull at the *MSTN* gene; (**b**) heterozygous bull at the *MSTN* gene.

In the investigated sample of Marchigiana beef cattle, 67 homozygotes normal (or wild-type) bulls (G/G), 11 heterozygote bulls (G/T), and no double-muscled homozygotes (T/T) at the *MSTN* gene were detected (Figure 1).

The analysis of the Hardy–Weinberg equilibrium showed that the Marchigiana genotyped sample resulted in equilibrium (0.75 < *p* < 0.90) (Table 1), as reported by Marchitelli et al. [28] in previous investigation on the same beef cattle breed.

Moreover, the chi-square test did not show significant differences between the genotype distribution at the *MSTN* locus of the present results and the ones reported by Lasagna et al. [30], as shown in Table S2. These data confirm that the frequency of heterozygotes at the *MSTN* gene is relatively low due to the breeding strategies applied to Marchigiana breed in the last decades, which do not allow *MSTN* homozygote bulls to be included in mating plans [31]. Therefore, the low frequency of the observed heterozygous genotype could be strictly related to the breeding strategy applied to the Marchigiana breed.

The average live weight of the Marchigiana bulls was 677.02 kg (range: 454–827 kg) at an average slaughter age of 632.45 days (range: 476–731 days). Bulls were slaughtered within the age of 24 months, according to the specification of the PGI “Vitellone Bianco dell’Appennino Centrale” mark.

In Table 2, the in vivo performance and carcass parameters, evaluated after slaughtering of 60 normal and 11 heterozygous *MSTN* animals are reported according to the analysis of covariance (Model 1-ANCOVA).

For the final live weight reached at slaughtering, no significant differences were observed between homozygous wild-type *MSTN* genotype and the heterozygous one. Despite the unbalanced dataset, heterozygous bulls showed an average final weight 17 kg higher than normal bulls. These findings may be ascribed to the ad libitum feeding regimen adopted during the trial and mainly based on concentrate, which allow hypertrophic beef cattle a better expression of their lower and late growth potential [32]. The light superiority in the live weight reached by heterozygous bulls, although not significant, agrees with other investigations carried out by Casas et al. [33] on Charolais and Belgian Blue x British Breed crosses, and by Gill et al. [34] on Angus cattle. On the contrary, Sarti et al. [35], in a small group of Marchigiana bulls, observed a lower final live weight in heterozygous bulls compared to normal bulls. This was probably due to the low number of genotyped bulls or to a low feed intake as a consequence of size reduction of the digestive tract in heterozygotes [7].

In addition, the ADG was not affected by genotype at the *MSTN* gene, resulting in quite a similarity in heterozygous and normal bulls. The daily gain weights observed herein were slightly lower than the daily gains observed by Casas et al. [33] in Charolaise and Belgian Blue x British Breed crosses wild-type or heterozygous at *MSTN* gene, and the ones reported by Vinet et al. [36] in a crossbred bulls’ population obtained by mating homozygotes mutated Blonde d’Aquitaine bulls to Holstein cows.

Regarding the results for the carcasses’ post mortem parameters (Model 1-ANCOVA), none of the parameters were affected by genotype. However, heterozygotes Marchigiana bulls reached 426.09 kg in carcass weight. Carcass traits of heterozygous bulls of French beef breeds [37], of Charolais and Belgian Blues crosses bulls [33], of British South Devon cattle breed [38], and of fifty-six Blond d’Aquitaine × Holstein crossbred calves [36] reached superior values compared to normal ones.

Although there are no significant differences between heterozygous and normal Marchigiana bulls, the carcass daily gain means suggest the effect of the mutation on this parameter, which should be supported by further research.

Heterozygous Marchigiana bulls reached 62.65% in dressing yield, while 60.96% was observed for normal bulls at the *MSTN* gene, although no significant difference between means was observed (*p* = 0.108). This trait contributes to quantifying the production efficiency and the economic benefits either for breeders or for the beef cattle industry. Therefore, Marchigiana heterozygotes showed better performance in dressing yield as compared to heterozygotes of Blond d’Aquitaine crosses, Charolaise, Piemontese, Asturiana de los Valles, and Limousine beef cattle breeds [33,36,39,40]. This may be ascribed to the increase in muscles mass, the fineness of the limb bones, and the reduction of the fifth quarter and of skin weight [7,32], reached in the Marchigiana breed through recent selection schemes applied by ANABIC [31].

The *MSTN* genotype did not affect the pH detected on carcasses at 45′ after slaughtering. Slaughter date as random term was significant (*p* < 0.001), but the differences between pH means of the two genotypes was not significant. The pH-45′ results suggest that bulls had not been subjected to stressful conditions before slaughter; usually greater pH differences are referenced 12–24 h after killing, mainly due to higher carcass temperatures in double-muscled heterozygous or homozygous animals [7].

Results of the genotype effect at the *MSTN* gene on steak dissection (Model 1-ANCOVA, HCW as covariate) are listed in Table 3.

Heterozygotes bulls showed a low, but not significant, incidence of bone portion at steak dissection, reflecting statements about the reduction of skeleton in *MSTN* mutated animals reported by several authors [7,10,19,30,32,35]. Steak samples from heterozygous animals showed a significantly higher proportion of other muscles (67.51%, *p* < 0.05) and a significantly lower proportion of subcutaneous and intermuscular fat (6.62%, *p* < 0.01) than normal bulls (60.33 and 10.37%, respectively). These results support the growth of muscular mass due to lack of myostatin protein function also in Marchigiana beef cattle, resulting in an increased number of muscle fibers (hyperplasia), fibers enlargement (hypertrophy), and a reduction in subcutaneous and intermuscular fat deposition [14,33,37,39,41].

Results of the genotypic effect at the *MSTN* gene on meat composition and its colorimetric profile of 78 Marchigiana bulls according to Model 3-ANOVA (except for drip loss, Model 2- ANOVA) are listed in Table 4.

Concerning the chemical composition of the LT in Marchigiana bulls, only the contents in fat and ashes were significantly affected by the genotype at the *MSTN* locus. The lowest level in fat observed in meat from heterozygote bulls (2.01%) confirms results obtained in previous research carried out in several beef cattle breeds [37,39,42]. The ashes content was significantly higher in heterozygous than normal bulls; values observed in the present investigation were slightly higher than those reported by Destefanis et al. [42] and Sarti et al. [43] in double-muscled bulls from Piemontese and Marchigiana breeds, respectively. The protein content of meat from heterozygote bulls exceeded 20%, although difference between the mean values of the two genotypes was not statistically confirmed. These analytical results support the different tissues’ distribution occurring in double-muscled beef cattle breeds and a typical leaner meat, as referenced in other investigations for animals carrying the double-muscled mutation [7,10,12,19,32,44,45].

It is noteworthy that the contents in protein, fat, and ashes observed herein in the heterozygous Marchigiana bulls at the *MSTN* locus fall within the ranges of the quality parameters required by the PGI specification “Vitellone Bianco dell’Appennino Centrale”, according to the Commission Regulation (EC) No 134/98 [17].

The meat’s water-holding capacity, measured through the evaluation of drip losses, did not highlight significant differences between meat from normal and heterozygous bulls, suggesting that the presence of one copy of mutated allele did not clearly interfere with the binding systems between muscle fibers. This is in agreement with a previous study from Reardon et al. [46] that reported for the first time associations among meat quality traits, including water-holding capacity, and polymorphisms in candidate genes, not comprising *MSTN*. Slightly higher water losses were observed in meat from G/T Marchigiana bulls than G/G ones, thus reflecting the more glycolytic muscle metabolism of heterozygous bulls [39].

The Model 3-ANOVA performed to test the effect of *MSTN* gene on colorimetric reflectance coordinates of meat from normal and heterozygous Marchigiana bulls did not detect any significant differences between means. Despite this, meat from heterozygous Marchigiana bulls showed high value in lightness (L*), thus confirming results from scientific literature reporting that meat of hypertrophic animals has a lighter color often associated to a lesser haem-pigment concentration than normal bulls [35,39]. L*, redness, and yellowness values observed for Marchigiana heterozygous meat were like the ones observed in Asturiana biotypes [47], at the same time-point (24 h post mortem). Moreover, the chroma value, an expression of the vividness of color, was quite similar to the one reported by Oliván et al. [39] in heterozygous Asturiana breed.

In Table 5, the different relative distributions of the muscular conformation and fat covering classes between 60 normal and 11 heterozygote bulls at *MSTN* gene are reported (71 carcasses evaluated out of 78 total).

The total chi-square test highlighted an overall significant difference among the assignment of carcasses to the three SEUROP classes between the two genotypes. Orthogonal contrasts showed a significantly higher frequency in the heterozygous group of class E than class U + R carcasses, whereas no significant difference between U and R classes was found (Table 5a). These findings confirm the better muscular conformation of hypertrophic bulls, due to a greater convexity of round, back, and shoulder muscular masses, as a consequence of the muscular hypertrophy observed and reported by several authors [7,18,30,34,36,37,38].

Concerning the amount of fat on the outside of the carcass and in the thoracic cavity, heterozygous Marchigiana bulls showed a higher, although not significant, frequency in class 2, with a slight fat cover, than normal ones (Table 5b). These findings confirm what has already been observed in previous research in several cattle breeds with hypertrophic animals, all characterized by a moderate carcass fat cover (mainly class 2 or 3) [7,30,34,36,37,38].

Overall, a future investigation with a larger sample size would be beneficial to validate the results observed in heterozygote Marchigiana bulls for in vivo and post mortem performances and meat quality parameters.

## 4. Conclusions

Our results confirm in the Marchigiana breed what has already been observed in other hypertrophied breeds. In particular, the mutation seems to influence important productive traits such as the final live weight, the dressing percentage, and the fat content of the meat.

The muscularity of heterozygous animals could be an interesting starting point to improve producing efficiency in Marchigiana beef cattle, to meet modern consumer needs and to face the increasing demand on products of animal origin.

Future applications linked to programmed matings and in-depth knowledge of the variants of *MSTN* gene in Marchigiana breed could also lead to developing new breeding schemes which will maintain heterozygotes in the breed, while limiting the diffusion of the mutate homozygous genotype to avoid the undesirable negative effects notoriously related to double-muscled phenotype.

## Figures and Tables

**Table 1:** Allelic and genotypic frequencies, and test for Hardy–Weinberg equilibrium (χ^2^) at *MSTN* locus.

*n*	Allele Freq. (%)		Observed Genotype Freq. (%)			χ^2^	*p*-Value
	G	T	G/G	G/T	T/T		
78	95.95	7.05	85.90	14.10	0.00	0.448	0.75 < *p* < 0.90

**Table 2:** *MSTN* genotype effect on in vivo and post mortem parameters of 71 Marchigiana bulls’ carcasses (standard error of Least Squares Means in parentheses).

Parameter ^1^	*MSTN* Genotype		Significance	
	G/G	G/T	Genotype	Cov ^2^
FLW	664.86 (7.36)	681.84 (17.34)	n.s.	***
ADG	1.07 (0.01)	1.10 (0.03)	n.s.	***
HCW	405.32 (5.32)	426.09 (12.53)	n.s.	*
CDG	0.65 (0.01)	0.69 (0.02)	n.s.	***
DY	60.96 (0.38)	62.55 (0.90)	n.s.	n.s.
pH-45′	6.79 (0.04)	6.97 (0.11)	n.s.	n.s.

^1^: FLW, final live weight (kg); ADG, average daily gain (kg); HCW, hot carcass weight (kg); CDG, carcass daily gain (kg); DY, dressing yield (%); pH-45′, pH measured within 45 min after slaughtering. ^2^: the covariate used in the model was the age at slaughtering (days) for all variables but pH-45′, for which hot carcass weight (kg) was used as covariate. n.s. = not significant; * = significant *p* < 0.05; *** = significant *p* < 0.001.

**Table 3:** *MSTN* genotype effect on steak dissection and on drip loss of Marchigiana bulls (standard error of Least Squares Means in parentheses).

Parameter (%)	*MSTN* Genotype		Significance	
	G/G	G/T	Genotype	Cov ^1^
Bone	13.08 (0.90)	9.94 (2.26)	n.s.	n.s.
LT muscle	15.91 (1.00)	16.40 (2.48)	n.s.	n.s.
Other muscles	60.33 ^a^ (1.25)	67.51 ^b^ (3.10)	*	n.s.
Fat	10.37 ^a^ (0.49)	6.62 ^b^ (1.20)	**	*

LT: *Longissimus thoracis* muscle ^1^: the covariate used in the model was the hot carcass weight (kg). Least squares means in the same row with different letter are significantly different (*p* = 0.05). n.s. = not significant; * = significant *p* < 0.05; ** = significant *p* < 0.01.

**Table 4:** *MSTN* genotype effect on analytical composition and colorimetric profile of meat from 78 Marchigiana bulls (standard error of Least Squares Means in parentheses).

Parameter (%)	*MSTN* Genotype		Significance
	G/G	G/T	Genotype
Water	73.41 (0.20)	73.69 (0.48)	n.s.
Protein	19.88 (0.11)	20.29 (0.27)	n.s.
Fat	3.04 ^a^ (0.16)	2.01 ^b^ (0.39)	*
Ashes	1.15 ^a^ (0.01)	1.26 ^b^ (0.03)	***
Drip Loss	1.05 (0.07)	1.21 (0.17)	n.s.
L*	41.04 (0.44)	42.31 (1.09)	n.s.
a*	24.54 (0.33)	24.47 (0.82)	n.s.
b*	7.06 (0.26)	7.40 (0.64)	n.s.
Chroma	25.61 (0.36)	25.64 (0.88)	n.s.

L*: lightness; a*: redness-greenness; b*: yellowness-blueness; chroma: (a*^2^ + b*^2^)^0.5^. Least square means in the same raw with different letter are significantly different (*p* = 0.05). n.s. = not significant; * = significant *p* < 0.05; *** = significant *p* < 0.001.

**Table 5:** Comparison (χ^2^ Test, df in square parentheses) between normal and heterozygous genotypes at *MSTN* gene for muscular conformation and fat covering according to SEUROP grid (relative frequencies are shown in parentheses). For muscular conformation, orthogonal contrasts (E vs. U + R and U vs. R classes) are reported.

	*MSTN* Genotype	
	G/G	G/T
**(a) Muscular conformation**		
Class E	1 (1.67%)	4 (36.36%)
Class U	49 (81.67%)	7 (63.64%)
Class R	10 (16.67%)	0 (0.00%)
Total Chi-square Test	χ^2^_[2]_ = 18.11; *p* < 0.001	
Orthogonal Contrasts:		
E vs. (U + R)	χ^2^_[1]_ = 17.09; *p* < 0.001	
U vs. R	χ^2^_[1]_ = 1.40; *p* < 0.237	
**(b) Fat covering**		
Class 2	34 (56.67%)	7 (63.64%)
Class 3	26 (43.33%)	4 (36.36%)
Total Chi-square Test	χ^2^_[1]_ = 0.19; *p* = 0.67	

Numbers within square brackets indicate the degree of freedom.

## Data Availability

The data presented in this study are available on request from the corresponding author.

## Supplementary Materials

The following supporting information can be downloaded at: https://www.mdpi.com/article/10.3390/ani12040518/s1, Table S1. Average chemical composition of ration ingredients on DM% basis (mean ± SD), Table S2. Genotype distribution at *MSTN* locus: comparison by χ^2^ test (df in square parentheses) between two different samples of Marchigiana beef cattle (relative frequencies are shown in parentheses) [30].
