# Supplementary material for: Effect of Myostatin Gene Mutation on Slaughtering Performance and Meat Quality in Marchigiana Bulls
Source: Animals (Basel). 2022 Feb 19;12(4):518. doi: 10.3390/ani12040518 (PMC8868461; doi:10.3390/ani12040518)
Supplement: Supplementary file 1 [file animals-12-00518-s001.zip › animals-1588427-supplementary.pdf]

Table S1. Average chemical composition of ration ingredients on dry matter (%) basis (mean ± standard deviation).

Parameter (%)	Hay<br>Mean ± Standard Deviation	Concentrate<br>Mean ± Standard Deviation
Moisture	11.73 ± 2.57	12.77 ± 1.05
Dry Matter	88.27 ± 2.57	87.23 ± 1.05
Crude Protein	8.96 ± 3.92	15.65 ± 1.98
Ether Extract	1.52 ± 0.50	3.03 ± 0.57
Crude Fiber	34.19 ± 4.04	5.83 ± 1.02
Neutral Detergent Fiber	65.90 ± 7.49	24.16 ± 3.41
Acid Detergent Fiber	45.44 ± 4.83	9.78 ± 1.07
Acid Detergent Lignin	9.12 ± 2.18	3.98 ± 1.29
Ash	8.55 ± 1.42	6.66 ± 1.92

Table S2. Genotype distribution at *MSTN* locus: comparison by χ^2 test (df in square parentheses) between two different samples of Marchigiana beef cattle (relative frequencies are shown in parentheses).

	N	*MSTN* Genotype	
		+/+	+/−
Present study	78	67 (85.90%)	11 (14.10%)
Lasagna et al. [30]	279	227 (81.37%)	52 (18.63%)
Chi-square Test		$X^2_{[1]} = 0.86$; $P = 0.35$	

Numbers within square brackets indicate the degree of freedom.
